# Site-Specific Conjugation for Fully Controlled Glycoconjugate Vaccine Preparation

**DOI:** 10.3389/fchem.2019.00726

**Published:** 2019-11-01

**Authors:** Aline Pillot, Alain Defontaine, Amina Fateh, Annie Lambert, Maruthi Prasanna, Mathieu Fanuel, Muriel Pipelier, Noemi Csaba, Typhaine Violo, Emilie Camberlein, Cyrille Grandjean

**Affiliations:** ^1^Université de Nantes, CNRS, Unité Fonctionnalité et Ingénierie des Protéines (UFIP), UMR 6286, Nantes, France; ^2^Université de Nantes, CNRS, Chimie Et Interdisciplinarité: Synthèse, Analyse, Modélisation (CEISAM), UMR 6230, Nantes, France; ^3^Department of Pharmacology, Pharmacy and Pharmaceutical Technology, Center for Research in Molecular Medicine and Chronic Diseases (CIMUS), School of Pharamacy, Health Research Institute of Santiago de Compostela (IDIS), University of Santiago de Compostela, Santiago de Compostela, Spain; ^4^Unité Biopolymères Interactions Assemblages Plate-Forme BIBS, INRA, Nantes, France

**Keywords:** glycoconjugate vaccine, cysteine mutagenesis, chemoselective ligation, protein conjugation, pneumococcal vaccine, thio/maleimide ligation

## Abstract

Glycoconjugate vaccines are formed by covalently link a carbohydrate antigen to a carrier protein whose role is to achieve a long lasting immune response directed against the carbohydrate antigen. The nature of the sugar antigen, its length, its ratio per carrier protein and the conjugation chemistry impact on both structure and the immune response of a glycoconjugate vaccine. In addition it has long been assumed that the sites at which the carbohydrate antigen is attached can also have an impact. These important issue can now be addressed owing to the development of novel chemoselective ligation reactions as well as techniques such as site-selective mutagenesis, glycoengineering, or extension of the genetic code. The preparation and characterization of homogeneous bivalent pneumococcal vaccines is reported. The preparation and characterization of homogeneous bivalent pneumococcal vaccines is reported. A synthetic tetrasaccharide representative of the serotype 14 capsular polysaccharide of *Streptococcus pneumoniae* has been linked using the thiol/maleimide coupling chemistry to four different Pneumococcal surface adhesin A (PsaA) mutants, each harboring a single cysteine mutation at a defined position. Humoral response of these 1 to 1 carbohydrate antigen/PsaA conjugates have been assessed in mice. Our results showed that the carbohydrate antigen-PsaA connectivity impacts the anti-carrier response and raise questions about the design of glycoconjugate vaccine whereby the protein plays the dual role of immunogen and carrier.

## Introduction

Surface exposed polysaccharides of bacterial pathogens are perceived as non-self by the host immune system. Therefore, they are often the target of a protective humoral immune response. Immunization using polysaccharides from capsulated bacteria has been introduced by Gotschlich in the late 1960s (Gotschlich et al., 1972). Capsular polysaccharides are typical T cell-independent type 2 antigens (Mond et al., 1995). This group of antigens is able to deliver prolonged and persistent signaling to the B cell through B cell receptor cross-linking. However, these vaccines fail to be active in children, the population which is the major target of the infectious diseases caused by pathogenic bacteria. A major breakthrough in the field has been later achieved by the development of the glycoconjugate vaccines (Rappuoli, 2018). A purified capsular polysaccharide or a fragment thereof or a synthetic oligosaccharide mimicking the antigenic determinants expressed by capsular polysaccharide is conjugated to a protein scaffold referred to as carrier protein. In this case, the protein moiety of the conjugate is processed by carbohydrate antigen-specific B cells after engagement of their B cell receptor. This leads to the presentation of peptides—T helper epitopes—in association with major histocompatibility complex of type II (MHCII) molecules to carrier peptide-specific CD4^+^ T lymphocytes. These T helper cells, in turn, stimulate the production of both plasma cells and memory B cells (Pollard et al., 2009). This vaccine strategy proved highly efficient even in infants. Consequently several vaccines active against meningococcus, streptococcus, or *Haemophilus influenza* type b have been launched (Berti and Adamo, 2018). Alternatively it has recently been shown that a carbohydrate epitope presented in the form of a glycopeptide by the MHCII molecules could strongly stimulate CD4^+^ T cells (Avci et al., 2011; Berti and Adamo, 2013). While both mechanisms probably coexist, this discovery might considerably impact the design of future glycoconjugate vaccines. Indeed, it has long been established that both length and density of the carbohydrate antigens on the carrier protein influence the immunogenicity of the conjugates in an interconnected manner. At a fixed sugar/protein ratio, the anti-carbohydrate antigen titers vary according to a bell curve as a function of density (Pozsgay et al., 1999). On the other hand, the observed optimum depends on the length of the antigen, this value being usually lowered when one increases the chain length (Anderson et al., 1989). However, if second mechanism has to be considered, the selection of the glycosylation sites is equally important. Along this line, Peng et al. have taken advantage of the propensity of flagellin to self-assemble in a supercoiled structure to selectively modify the sole lysines exposed to the solvent and thus preserving the protein properties to activate immune response (Peng et al., 2018). Stefanetti et al. recently prepared a series of glycoconjugates made of CRM_197_ and *Salmonella O*-antigen as the carrier protein and the carbohydrate antigen, respectively (Stefanetti et al., 2015). An average of one up to four *O*-antigen chains per protein was introduced at controlled positions. The *O*-antigen chains were randomly linked to surface accessible glutamic/aspartic acid or lysine residues or at more defined sites upon exploiting the kinetically favored reactivity of lysines having the lowest p*K*_a_ (Crotti et al., 2014; Matos et al., 2018), the rarity of surface exposed tyrosine selectively activated (Hu et al., 2013; Nilo et al., 2014), the transglutaminase catalyzed modification of a lysine (Nilo et al., 2015b) or upon designing stapled conjugate from a reduced disulfide bond. This study was useful to demonstrate that the conjugation site plays a role in determining the immunogenicity. However, the tested formulations still contained heterogeneous mixtures of conjugates since the derivatization processes remain largely empirical. Moreover, further discrepancies arose from structural differences among the linkers used in the study although the same strain promoted azide-alkyne cycloaddition reaction was applied for the preparation of every conjugate. Such biases do not allow an unequivocal interpretation of observed results. Yet recent progress made in unnatural amino acid incorporation (Quast et al., 2015), protein glycan coupling technology (PGCT) (Ma et al., 2018) or site-selective mutagenesis (Grayson et al., 2011) offer unique opportunities to access fully defined glycoconjugate vaccines and further elucidate the relationship between carbohydrate antigen/carrier protein connectivity and immunogenicity. We report herein the preparation and the characterization of homogeneous bivalent pneumococcal conjugates. A synthetic tetrasaccharide derived from *Streptococcus pneumoniae* serotype 14 capsular polysaccharide equipped of a maleimido-functionalized spacer arm at its reducing end has been site-specifically attached to four different cysteine mono-mutants of the Pneumococcal surface adhesin A (PsaA).

## Results and Discussion

### Conjugate Design, Synthesis, and Characterization

**Figure 1:**
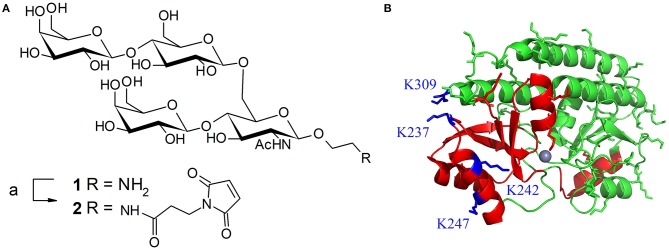
**(A)** Structure of tetrasaccharide antigen **1** from *S. pneumoniae* serotype 14 capsule and its activation with a maleimide linker; reagents and conditions: (a) 3-maleimidopropionic acid *N*-hydrosysuccimide ester (1.45 equiv), DIEA (2 equiv), DMF, RT, overnight, 58%. **(B)** Ribbon diagram of PsaA (green). Regions containing known Th and B-epitopes are colored in red. Lysine side-chain are represented in green except the four lysines targeted for mutagenesis (in blue). Representation based on the 1PSZ PDB file, with a resolution of 2.0 Å (Lawrence et al., 1998).

Pneumococcal infections are still a leading cause of mortality worldwide. Available prophylactic pneumococcal glycoconjugate vaccines induce capsule-specific memory B-cells and IgG capable to prevent colonization and disease (Jochems et al., 2017). Vaccine effectiveness is considerably improved by increasing the valency e.g., from 7 up to 13 serotypes (van der Linden et al., 2016). However, inclusion of serotype-independent immunogens able to control pneumococcal carriage to these vaccines has been identified as an appealing strategy (Jochems et al., 2017). PsaA is a nasopharyngeal colonization factor which is expressed by more than 99% of pneumococcal strains in a highly conserved form (Rajam et al., 2008a). These features have thus designed PsaA as a possible protein immunogen candidate (Wang et al., 2010; Gor et al., 2011; Olafsdottir et al., 2012; Lu et al., 2015). Concomitant administration of PsaA with PCV7 was accompanied with reduced colonization in a murine model (Whaley et al., 2010) and its protective effect in association with a panel of pneumococcal protein immunogens later assessed in phase I clinical trials (Schmid et al., 2011; Entwisle et al., 2017). Moreover, the successful use of PsaA both as an immunogen and a carrier protein PsaA by several laboratories including ours in mice models further encouraged us to select it as a model protein (Lin et al., 2010; Chen et al., 2016; Prasanna et al., 2019). Mature PsaA (mPsaA) i.e., PsaA deprived from its signal peptide, was therefore conjugated to the tetrasaccharide β−D−*Galp*−(1 → 4)−β−D−*Glcp*−(1 → 6)−[β−D−*Galp*−(1 → 4)]β-d-GlcpNAc **1** (referred to as Pn14TS) (Figure 1A). This easily synthesized tetrasaccharide is the minimal structure from the capsule of serotype 14, one of the prevalent pneumococcal serotype which has developed high antibiotic resistance (Yahiaoui et al., 2018), able to induce functional antibodies (Abs) (Safari et al., 2008). We observed that while high anti-mPsaA Ab titers were induced in mice immunized with mPsaA alone, this response was much lower when mice where immunized with a conjugate whereby Pn14TS was randomly coupled to surface-exposed lysine side-chains of mPsaA at a 5.4:1 average value for carbohydrate:protein ratio (Prasanna et al., 2019). This observation might result from mPsaA B epitope masking due to Pn14TS conjugation on the protein, immune-dominance of antigenic determinants expressed by Pn14TS over the mPsaA B epitopes or different processing of free vs. conjugated mPsaA. Developing an access to homogeneous Pn14TS-mPsaA conjugates will therefore provide tools to address these issues and document structure-immunogenicity relationships useful for future pneumococcal vaccine design.

mPsaA is a 32.4 kDa-protein which corresponds to amino acids 21-309 of PsaA sequence. mPsaA essentially adopts alpha-helical secondary structures and contains 37 lysines, most of them accessible for conjugation (Figure 1B) (Couñago et al., 2014). Interestingly, mPsaA does not contain any cysteine residue which makes site-selective cysteine mutagenesis an attractive approach to envision homogenous mPsaA conjugate synthesis (Grayson et al., 2011). Previous studies based on secondary structure predictions, endopeptidase site analyses and *in silico* MCHII peptide-binding affinity screening helped identifying a panel of 24 putative PsaA T-helper epitopes. Three out of them proved to be able to provoke Th cell proliferation: PsaA^67−82^, PsaA^199−221^, and PsaA^231−268^. The last one was deduced from three potent overlapping 15-mer peptides among which sequence 243–257 was the most potent (Singh et al., 2014). Identification of PsaA B epitopes has also been carried out using a phage display peptide library and monoclonal Abs. Two sequences in the region 132–146 and 253–267 showed promises for their immunogenicity in mice noticeably for reducing carriage and colonization (Srivastava et al., 2000; Johnson et al., 2002). Further studies have demonstrated that the sequence PsaA^251−278^ was involved in PsaA-mediated adherence of *S. pneumoniae* to epithelial cells (Romero-Steiner et al., 2006; Rajam et al., 2008b). In view of these data, we elected to mutate C-terminal lysine 309 located in an apparently non-relevant region for immunity into a cysteine. The preparation of three additional cysteine mutants at K237, K242, and K247 i.e., within or close to the potent T-helper epitope was also envisaged (Figure 1B).

**Figure 2:**
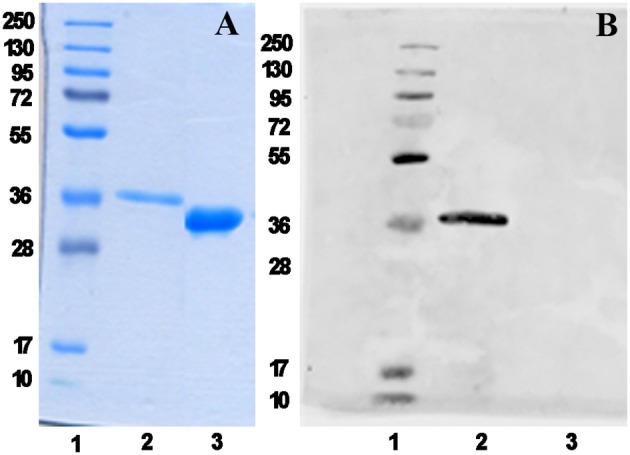
mPsaA K309C characterization as a representative example. Analysis of histidine tag removal from mutant mPsaA K309C by SDS-PAGE **(A)** and by Western blot **(B)**. Lane 1: unstained protein marker; Lane 2: tagged mPsaA K309C; Lane 3: purified mPsaA K309C after removal of poly-histidine tag.

We have recently reported the production in *Escherichia coli* BL21(DE3) of the mPsaA *N*-terminated by a poly-6-histidine tag sequence to facilitate its purification by immobilized-Ni affinity chromatography plus a Tabacco Etch Protease (TEV) specific cleavage site to remove the tag after purification (Prasanna et al., 2019). mPsaA production level has been increased by 5–6 fold upon adopting time/temperature/induction conditions reported by Laurentis et al. and replacing the LB by the TB growth medium (Supplementary Figure 1 and Supplementary Table 1) (Larentis et al., 2011). The four mutants have been produced accordingly after having introduced every desired mutation in the original sequence using the QuickChange method. Culture have been carried out at a 250 ml scale and yielded about 19 mg of each mutant after purification and histidine tag removal (Figure 2).

**Figure 3:**
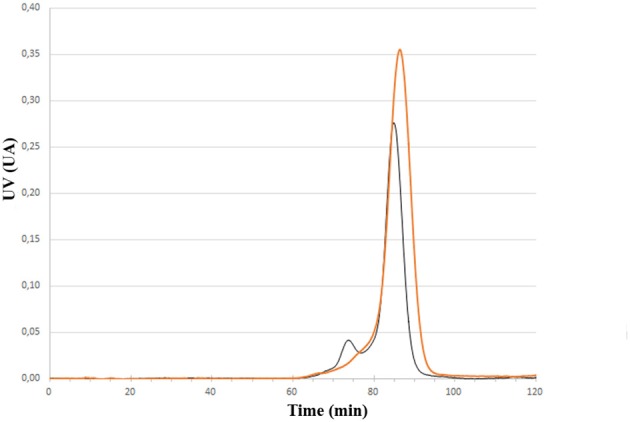
Comparison of size exclusion chromatography profile of Pn14TS-mPsaA K247C (red trace) and Pn14TS-mPsaA K309C (Black trace) conjugates. Column HiLoad 15/600 Superdex™ 75 pg (GE Healthcare) column and PBS 0.1 M, pH 7.3 as eluent.

**Figure 4:**
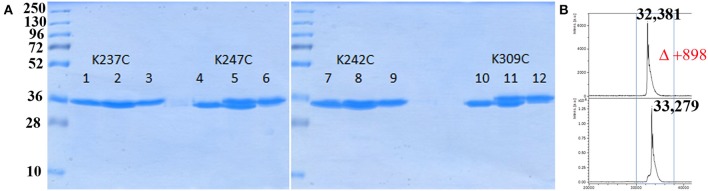
Analysis of bioconjugation efficiency by SDS PAGE and mass spectrometry. **(A)** Comparison of each mPsaA mutant before, mix of before and after, and after conjugation. Lanes 1–3: mPsaA K237C; Lanes 4–6: mPsaA K247C; Lanes 7–9: mPsaA K242C; Lanes 10–12: mPsaA K309C. Two micrograms protein sample/lane, 12% SDS-PAGE, 100 V, 2 h; **(B)** MALDI MS spectrum of mPsaA K309C (top spectrum) and Pn14TS-mPsaA K309C (bottom spectrum).

Having the four mutants in hand we next examined their respective conjugation with the carbohydrate antigen. To this aim, known tetrasaccharide **1** (Prasanna et al., 2019) was derivatized with a maleimide linker to provide **2** in 58% yield following RP-HPLC purification (Figure 1A). The four mutants were treated separately with DTT to reduce any inter-protein disulfide bond which might have formed during their preparation and further reacted with no more than 5 equivalent of **2** in degassed PBS, pH 7.0 at room temperature overnight. Each conjugate was then subjected to gel filtration purification to remove excess tetrasaccharide (Figure 3) and freeze-dried for storage. Gel filtration chromatography profiles of conjugates associated to mPsaA K237C, K242C, and K247C were very similar and essentially composed of a single peak. A second unidentified peak which is eluted earlier is apparent in the Pn14TS-mPsaA K309C profile (Figure 3). We first thought that it corresponds to a dimer of the mPsaA K309C whose formation had been favored due to a greater exposure of the cysteine at the C-terminus. However, mass spectrometry experiments ruled out this hypothesis. Effectiveness of the conjugation was checked by gel electrophoresis which uniformly showed that spots are shifted toward higher molecular weight, compatible with the attachment of a single tetrasaccharide (Figure 4A). These results were confirmed by MALDI mass spectrometry experiments which indicate that parent mutant mPsaA proteins were incremented by 896–906 mass unit (calculated +901 Da) within the error range mass measurements (Figure 4B). In the end homogenous 1:1 carbohydrate antigen/carrier protein K237C, K242C, K247C, K309C conjugates have been obtained in 55, 36, 44, and 30% yield, respectively, with a purity superior to 90%.

### Humoral Response Evaluation

**Figure 5:**
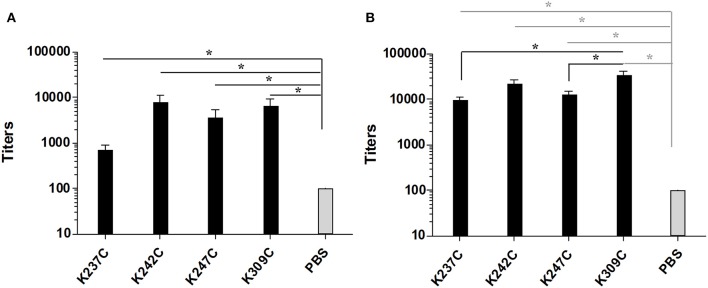
Titers of anti-mPsaA (coated on microtiter plates) IgG Abs of mice immunized with Pn14TS-mPsaA K237C, K242C, K247C, K309C, PBS 1 week after the 2nd (J21) **(A)** and the 3rd (J35) immunization **(B)**. The serum samples data presented as geometric mean titer ± standard deviation of five mice per group. Statistical analysis was performed using one-way ANOVA with Tukey analysis for multiple comparisons. Statistical difference between the groups is ^*^P < 0.05.

Groups of five C57/BL6 mice were immunized thrice at 2 weeks interval with each of the four conjugates and PBS as a negative control formulated in chitosan and Ribi as adjuvant. The antibody response against both mPsaA and capsular polysaccharide of *S. pneumoniae* serotype 14 (CP14), was then determined by ELISA assays 1 week after the second and third immunization (i.e., on Days 21 and 35). The IgG response raised against CP14 by the conjugates was very low and not significant compared to the negative control group (data not shown). This response was equally low when we used Pn14TS as the coating antigen indicating that the observed results were not due to a lack of recognition of CP14 by anti-Pn14TS Abs. Anti-CP14 IgM response was also determined after the second and the third immunizations. Low titers of anti-CP14 IgM Abs could be measured in the sera of mice for any of the tested formulations after the second immunization (Supplementary Figure 2A). Highest titers of anti-CP14 Abs seem to be induced by Pn14TS-mPsaA K247C conjugate in comparison with other conjugates and PBS. Pn14TS-mPsaA K309C gave rise to the weakest response comparable to that induced by PBS. However, these tendencies were not statistically significant. Anti-IgM responses further diminished after the boost although the expected IgM to IgG switch was not observed (Supplementary Figure 2B). In fact, we and other experienced in the past that Pn14Ts was able to efficiently mimic native CP14 (Safari et al., 2008; Kurbatova et al., 2017; Prasanna et al., 2019). However, as mentioned in the introduction, it is assumed that the level of the anti-carbohydrate antigen response depends on intricate related parameters such as carbohydrate antigen length, carbohydrate antigen:carrier (S/P) ratio and administered sugar dose (Pozsgay et al., 1999). For examples, significant humoral response has been previously observed against Pn14TS: this hapten being used at a S/P ratio of 4, at a 2.5 μg dose using adipic acid coupling chemistry and CRM_197_ as the carrier (Mawas et al., 2002); at a S/P ratio of 4.8 or 6, at a 2.5 μg dose, using squarate coupling chemistry and CRM_197_ as carrier protein (Mawas et al., 2002; Safari et al., 2008); at a S/P ratio of 11, at 1.25–10 μg dose, using squarate chemistry and BSA as carrier; at a S/P ratio of 5.4, at 3 μg dose using thio/maleimide coupling chemistry and PsaA as carrier protein. Testing of Pn14TS at a S/P of 1 is unprecedented. Absence of anti-CP14 IgG response might be circumvented upon increasing the length of the antigen. Effective response was nicely observed when a dodecasaccharide (corresponding to 3 × Pn14TS units), conjugated to CRM_197_ was used in a 1:1 carbohydrate antigen/carrier protein ratio (Safari et al., 2008). Increasing the administered dose could also been envisaged. A very low amount of carbohydrate antigens (0.5 μg/dose/mouse) was indeed used for immunizations. This value was chosen to keep the amount of injected mPsaA equal to 25 μg/dose/mouse and remaining coherent with our previous experiments (Prasanna et al., 2019). The adopted strategy was nevertheless applicable to the investigation of the anti-mPsaA response. IgM Ab response was absent in all tested sera in agreement with our previous findings (data not shown) (Prasanna et al., 2019). Contrasting with these results a significant response was observed in the secondary sera of mice immunized with the conjugates in comparison with the control group (*P* < 0.05) (Figure 5A). Level of anti-mPsaA IgG titers was further raised up to 1/8,000–1/64,000 for all sera after the third immunization. The highest titers were observed in sera of mice immunized with Pn14TS-mPsaA K242C and K309C. Noticeably, anti-mPsaA IgG Abs induced by the later conjugate was significantly higher than those induced by Pn14TS-mPsaA K237C or Pn14TS-mPsaA K247C (*P* < 0.05) but not Pn14TS-mPsaA K242C (Figure 5B). We have recently shown that mPsaA was highly immunogenic in mice while introduction of only five tetrasaccharide haptens by classic lysine random conjugation severely impaired its immunogenicity (Prasanna et al., 2019). Maintaining the protective properties of the mPsaA epitopes is of paramount importance if a dual role of both vaccine immunogen and carrier for carbohydrate antigens is envisaged. Dual anti-Group B *Streptococcus* glycoconjugate vaccines have been prepared using transglutaminase or tyrosine-directed conjugation technology. This coupling strategy was shown to preserve the antigenicity of the *Streptococcus* proteins used as the carriers (Nilo et al., 2014). Immunogenicity of the conjugates with defined connectivity was essentially comparable to that observed for conjugates obtained upon conjugation of surface-exposed lysine side-chains (Nilo et al., 2015a,b). In a different study, PGCT was applied to the controlled transfer of *E. coli* O157:H7 *O*-polysaccharide to the asparagine residue amino acid sequence (DQNAT)_4_ introduced at the C-terminus of the maltose-binding protein used as carrier protein model (Ma et al., 2018). The glycosylation had slight interference on the global anti-MBP response. However, for the first time it has been shown that the carbohydrate antigen decreased the response against the peptides containing or adjacent to the polysaccharide but not against peptide at distal site. Our results are in agreement with these data. Immunogenicity of mPsaA is globally conserved but finely tuned by the grafting of a single carbohydrate antigen. The highest anti-mPsaA response is observed when Pn14TS is introduced at position 309 of mPsaA i.e., in a peptide segment distant from the identified T-helper epitope and not when it is introduced at position 237, 242, or 247. This result makes sense if mPsaA B-cell epitopes and Pn14TS antigen compete for the assistance of the same T-helper epitopes. CRM_197_ remains the best carrier protein develop so far for use in humans. Structural alteration of the epitopes expressed by CRM_197_ during conjugation and detoxification processes have been proposed to explain its superiority as a carrier protein over diphtheria toxoid or tetanus toxoid (Pecetta et al., 2015). Beyond, if one cannot improve the response against one antigen at the detriment of the response against another equally important one then the strategy consisting in using a protein with dual role of antigen and carrier is questionable. Consistent with this assumption, the weakest anti-Pn14TS response was measured in sera of mice immunized with the Pn14TS-mPsaA K309C conjugate. However, further studies are required to confirm this tendency since the anti-CP14 titers were weak and limited to IgM Abs. In a study based on the use of group B streptococcal type III polysaccharide, it has been calculated that approximately eight repeat units of a polysaccharide branched to a T-helper peptide could be presented in complex with a MCHII molecule to a T-cell receptor (TCR) (Avci et al., 2011). We herein elected to work with a short tetrasaccharide antigen. It is conceivable that a TCR can recognize both our small carbohydrate antigen and part of the T-helper peptide sequence even though this is not yet demonstrated. Access to longer synthetic oligosaccharides is more demanding from the synthetic aspect and not necessarily associated with improved antigenicity (Safari et al., 2008). Also the use of purified CP will be accompanied by a loss of conjugate homogeneity while complexing its immune investigation. However, administration of a higher dose of sugar might improve the response against the Pn14TS and allow assessment of the variation of both anti-sugar and anti-protein responses. Alternatively, such study could be envisaged upon coupling not one but several carbohydrate antigen at controlled positions. Further work is also needed to assess the protective properties of the Abs and to investigate in detail the B-cell response, in particular which part of the protein is targeted by the humoral response.

## Conclusion

The development of technologies based on site-directed or site-specific conjugation for example relying on cysteine mutagenesis as exemplified in this study can lead to fully characterized glycoconjugate vaccines while ensuring higher batch-to-batch reproducibility. Moreover, it offers unique opportunity to study structure-immunogenicity relationship while unraveling molecular aspects of the immune response and giving rise to an optimized generation of glycoconjugate vaccines.

## Experimental Section

### Mutagenesis

Mutations K237C, K242C, K247C, and K309C have been performed by PCR amplification Phusion polymerase (Thermo Scienyific), followed by DpnI digestion using the following primers: K237C forward 5′-GCACCCCGGAGCAAATCTGCACCCTGGTGGAAAAGC, reverse 5′-GCTTTTCCACCAGGGTGCAGATTTGCTCCGGGGTGC; K242C forward 5′-CAAATCAAAACCCTGGTGGAATGCCTGCGTCAGACCAAAGTTCCG, reverse 5′-CGGAACTTTGGTCTGACGCAGGCATTCCACCAGGGTTTTGATTTG; K247C forward 5′- GAAAAGCTGCGTCAGACCTGCGTTCCGAGCCTGTTCGTG, reverse 5′-CACGAACAGGCTCGGAACGCAGGTCTGACGCAGCTTTTC; K309C forward 5′-GGAGGGTCTGGCGTGCTAAGGATCCGGC, reverse 5′-GCCGGATCCTTAGCACGCCAGACC CTCC. The plasmids obtained are then transformed into competent XL1 blue bacteria. For each mutation, extraction of the plasmid (Quiaprep®Miniprep from Quiagen) on three different clones is carried out according to manufacturer protocol, and insertion of the mutations checked by sequencing.

### Mutant mPsaA Expression

The validated plasmids have been used to transform *E. coli* BL21 (DE3) strains for expression of the different mutants. The expression of mPsaA mutant proteins was performed according to the following protocol. Briefly, 250 mL of fresh TB medium with 100 μg/ml ampicillin were inoculated with 0.5 mL of overnight pre-culture and left to grow at 37°C, 180 rpm until they reach the exponential phase (approximately OD of 0.6/0.7 is reached) and at this point the HtTEV-mPsaA expression in the cultures were induced with IPTG (0.1 mM final concentration). The cultures were left to grow for another 16 h at 25°C and 180 rpm. The cells were harvested at 10,000 rpm. The pellet were re-suspended in 25 ml cold lysis buffer (50 mM NaH_2_PO_4_, 150 mM NaCl, pH 8.0 + 5 mM Imidazole + 1 mM PMSF) + 1 μg/mL DNAse, and 1 mg/mL of lysozyme incubated at 4°C under stirring during 30 min and were subjected to sonication (7 min, 50% amplitude, pulse of 5 s ON/OFF) while they were kept in ice, and the cell debris were removed by centrifugation (30 min, 13,000 rpm). The obtained supernatant was filtered (0.45 μm) and incubated with 0.5 ml of preconditioned Ni NTA beads for 1 h on rotating disk at 4°C, 12 rpm. Unbound fraction was collected, and the beads were washed with a lysis buffer containing 2–10 mM imidazole. The elution was performed with a lysis buffer containing 300 mM imidazole. The eluted fractions were subjected to SDS-PAGE to identify the fractions containing target protein. Later, the eluted fractions, containing the target protein (100 mg/L of culture), were pooled and dialyzed against water at 4°C.

### Removal of Poly-6-Histidine Tag and Western Blot Analysis

The enzymatic cleavage of the histidine tag from mutants was performed using a 5:1 HtTEV-mPsaA/AcTEV^TM^ protease ratio in the reaction buffer (50 mM Tris-HCl (pH 7.6), 1 mM EDTA, 1 mM DTT), for 16 h at 20°C, without stirring. The crude reaction mixtures were then dialyzed against water and incubated with the Ni NTA beads for the separation of the cleaved mPsaA mutants from the histidine tag, parent mPsaA mutants if not entirely digested and the tagged TEV protease. The eluate was collected and was subjected to SDS-PAGE and western blot analyses to confirm the removal of the histidine tag and assess their purity.

For the western blot, the vertical SDS-PAGE was carried out using 12% acrylamide gels with the loadings of the reaction mixture and tagged mPsaA mutants as controls using the Bio-rad system. The proteins were transferred to a nitrocellulose membrane at 150 mA for 90 min. For the antibody probing, the nitrocellulose membrane was initially blocked with TBS containing 5% skim milk and 0.1% tween 20 for 1 h at RT. A primary antibody to mouse anti-poly histidine (Sigma H1029, diluted 1:1,000), was applied to the membrane and incubated for 16 h at RT with agitation. The membranes were washed thrice with PBS containing 0.1% tween 20 and incubated with secondary antibody (Goat anti-Mouse IgG (H+L) Secondary Antibody, Alexa Fluor 680) for 1 h at RT on a shaker. Membranes were subjected to a final wash with PBS, and the detection was performed using an Odyssey CLx scanner (LI-COR) at 700 nm.

### 2-(*N*-3-maleimidopropanoyl)ethyl(β−*d*−*galactopyranosyl*)−(1 → 4)−(β−*d*−*galactopyranosyl*)−(1 → 6)−[(β−*d*−*galactopyranosyl*)−(1 → 4)]−2−*deoxy*−2−*acetamido*−β-d-glucopyranoside 2

To a solution of 2-amino-ethyl (β−D−*galactopyranosyl*)−(1 → 4)−(β−D−*galactopyranosyl*)−(1 → 6)−[(β−D−*galactopyranosyl*)−(1 → 4)]−2−*deoxy*−2−*acetamido*−β-d-glucopyranoside **1** (17.5 mg, 0.023 mmol, 1 equiv) in DMF were successively added 3-maleimido-propionic acid succinimidyl ester (9 mg, 1.45 equiv) and DIEA (8.30 μL, 2 equiv) at RT. The reaction mixture was stirred at RT overnight, diluted in water and freeze-dried. The crude residue was purified by RP-HPLC to provide **2** (12.2 mg, 58% yield); *R*_f_ 0.39 (nBuOH/EtOH/H_2_O 5:5:3); ^1^H NMR (400 MHz, D_2_O): δ 6.85 (s, 2H), 4.52 (d, *J* = 8.2 Hz, 1H), 4.51 (d, *J* = 7.8 Hz, 2H), 4.43 (d, *J* = 7.8 Hz, 1H), 4.26 (dd, *J* = 1.9, 11.7 Hz, 1H), 3.99–3.88 (m, 4H), 3.84–3.62 (m, 20H), 3.60–3.55 (m, 1H), 3.54–3.48 (m, 2H), 3.36 (ddd, *J* = 2.5, 7.8, 9.8 Hz, 1H), 3.28 (t, *J* = 4.1 Hz, 2H), 2.48 (t, *J* = 7.0 Hz 2H), 2.00 (s, 3H); ^13^C NMR (100 MHz, D_2_O): δ 174.7, 173.5, 172.6, 134.5, 103.0, 102.8, 102.5, 101.2, 78.5, 77.9, 75.4, 75.3, 74.7, 74.3, 73.5, 72.7, 72.6, 72.6, 72.4, 71.0, 71.0, 68.9, 68.6, 68.2, 67.4, 61.1, 61.0, 60.2, 55.1, 39.3, 34.7, 34.5, 22.3; HR-ESI-MS: *m/z* Calcd for C_35_*H*_55_*N*_3_*O*_24_[*M*+*Na*]^+^ 924.3073, found 924.3088.

### Glycoconjugate Syntheses

mPsaA mutants proteins are rehydrated with ultrapure water to reconstitute 50 mM phosphate buffer. The pH are tested and adjusted to pH 8 with NaOH 0.1 M if necessary. mPsaA solutions are then treated with DTT (1.6 mg/mg of protein). Solutions are mixed first by flush and next let in rotary shaker at room temperature for 15 min. DTT is eliminated with desalting column (Zeba Spin, 7 MWCO) prior equilibrated with *degassed* pH 7 PBS buffer using swing centrifuge. Protein contents are quantified using Nanodrop spectrophotometer at 280 nm prior their conjugation. To a solution of mPsaA mutants (4.8–7.2 mg) in degassed 40 mM PBS, pH 7.0 (1.8–2.4 mg/mL), was added **2** (5 equiv) dissolved in water (1 mg/mL), and the resulting mixture stirred overnight and then purified by gel filtration using a HiLoad 15/600 Superdex™ 75 pg (GE Healthcare) column and 0.1 M PBS, pH 7.3 as eluent at 0.8 ml/min. Collected fractions were concentrated by centrifugal concentrators (cut-off 3 MWCO) (Vivaspin; 1 h 7,000 g 4°C) and then freeze-dried to give the corresponding conjugates which were analyzed for identity and purity by gel electrophoresis and mass spectrometry.

### Immunizations

The conjugates (40 μl of a 5 mg/ml solution in PBS), were first formulated with a mixture of chitosan/poloxamer/TPP (1:1:1) (6 mL) under stirring (600 rpm) for 30 min. The particles were concentrated by centrifugation at 12000 RCF for 12 min at 15°C, using 10 μL of glycerol bed. After the centrifugation, the pellet in the bottom is carefully collected and re-suspended in PBS (50 μL). The Groups of 5 male C57/BL6JRj (5 week old) mice were injected sub-cutaneously (s.c.) with PBS, Pn14TS-mPsaA K237C, Pn14TS-mPsaA K242C, Pn14TS-mPsaA K247C, or Pn14TS-mPsaA K309C (25 μg protein/dose-−0.5 μg tetrasaccharide/dose) diluted with 50 μL of RIBI in PBS. The mice were immunized at day 0, 14, and 28. The sera were collected on days 21 and 35. Sera were stored at −80°C.

### Measurement of Humoral Response

The Ab responses induced upon immunizations were assessed 1 week after the second and the third injections by ELISA. mPsaA and capsular polysaccharide serotype 14 (CP14) (Alliance Bio Expertise), were used as coated antigens to define the anti-mPsaA or anti-CP14 Ab titers. mPsaA (0.1 μg/well) in 10 mM PBS, pH 7.3 (100 μL/well), was coated on 96 wells microtiter plates Nunc Maxisorp (ThermoFisher Scientific) plates overnight at 4°C. CP14 (1 μg/well) was coated for 48 h at 4°C in 10 mM PBS, pH 7.3 (100 μL/well). Plates were then washed with PBS 0.05% Tween 20 (3 × 200 μL), saturated using PBS containing 10% skimmed milk at 37°C for 2 h, then washed using PBS Tween 20 (PBST, 50 mM Tris, 150 mM NaCl, 0,1% Tween 20) (3 × 200 μL). Series of dilution of sera in PBS containing 10% skimmed milk (100 μL/well), were incubated at 37°C for 2 h. Plates were then washed with PBST (3 × 200 μL) and then incubated with goat anti-mouse IgG(H+L)-horse radish peroxidase-labeled conjugate (CliniSciences) used as secondary Ab at a dilution of 1/6,000, for 1 h at 37°C and further washed with PBST (5 × 200 μL). The enzyme substrate, o-phenylenediamine dihydrochloride (100 μL at 0.4 mg mL^−1^) in 0.1 M sodium citrate (pH 5.2), containing 0.02% hydrogen peroxide, was added to each well and the plate incubated for 20 min at RT in the dark. The reaction was terminated by adding 3 M HCl (1,000 μL per well), and the A492 was read in an Infinite M1000 spectrophotometer (TECAN). The Ab titer was defined as the dilution of immune serum that gave an OD (405 nm) at least twice that observed with pre-immune serum.

## Data Availability Statement

All datasets generated for this study are included in the article/Supplementary Material.

## Ethics Statement

The animal study was reviewed and approved by ethical permit number from Comité d'Ethique en Expérimentation Animale (CEEA): 7897.

## Author Contributions

CG designed the research and wrote the manuscript with AP, AD, and AL. MPr developed the route for the production of PsaA under EC, NC, and CG supervision. AP and AD produced the PsaA mutants under MPi, TV, and EC guidance. AL and CG carried out the conjugation step and the purification of the conjugates. AP and AF performed the ELISA assays. MF assisted in mass measurements.

### Conflict of Interest

The authors declare that the research was conducted in the absence of any commercial or financial relationships that could be construed as a potential conflict of interest.
