# Supplementary material for: Site-Specific Conjugation for Fully Controlled Glycoconjugate Vaccine Preparation
Source: Front Chem. 2019 Nov 1;7:726. doi: 10.3389/fchem.2019.00726 (PMC6839274; doi:10.3389/fchem.2019.00726)
Supplement: Supplementary file 1 [file Data_Sheet_1.PDF]

Supplementary Material

CONTENTS

General 3

Optimization of mPsaA production 3

Anti-CP14 IgM antibody response 5

^{1}H and ^{13}C NMR spectra of tetrasaccharide **2** 6

General

The antibiotic ampicillin was purchased from PanReac Applichem. The LB or TB culture medium was purchased from VWR. The isopropyl thiogalactoside (IPTG) used in the induction of protein was obtained from Alfa-Aesar. The Ni NTA beads used in the affinity chromatography for the purification of the mPsaA were obtained from Macherey-Nagel. The linker 3-maleimidopropionic acid succinimidyl ester was obtained from Interchim. The RIBI adjuvant was purchased from Sigma-Aldrich.

RP-HPLC separation was performed on a Uptisphere Strategy 100 Å C18HQ (Interchim, France) (5 µm, 21.2 × 250 mm) column at a flow rate of 6 mL min^{-1} with ELSD and UV (225 nm) detection. Gradient: 0% B for 5 min, 0–5% B over 5 min, 10–80% B over 30 min, 80%B for 5 min; Solvent system A: H_2O; solvent system B: MeOH.

NMR experiments were performed at 400.13 MHz using Bruker Avance 400 MHz spectrometer equipped with a DUAL+ $^{1}H/^{13}C$ ATMA grad 5 mm probe. Assignments were performed by stepwise identification using COSY, and HSQC experiments using standard pulse programs from the Bruker library. Chemical shifts are given relative to external TMS with calibration involving the residual solvent signals.

mPsaA mass spectra were determined on an Autoflex III MALDI-TOF/TOF spectrometer (Bruker Daltonics) in positive ionisation modes and with a linear detection. 75 pmol of each sample was deposited on the MALDI target plate and were mixed with sinapinic acid as the matrix (10 mg mL−1; H_2O–CH_3CN–TFA, 50:50:0.1). The mass spectrometer was calibrated with a standard of BSA (15 pmol on target) on dimer and different charge states of the protein.

Optimization of mPsaA production

Two distinct protocols combining two different culture broth with different incubation time and temperature were compared: incubation at 37°C during 4 hours after induction with 1 mM IPTG (Protocol 1) or incubation at 25°C during 16 hours after induction with 0.1 mM IPTG (Protocol 2) (Larentis et al, 2011)[1]. In addition, effect of LB growth medium was compared to that of highly enriched Terrific Broth (TB) medium.

The four productions were made in 20 mL of each medium (LB and TB) and were induced by adding the desired amount of IPTG when the OD at 600 nm was near 0.8. After incubation time, 600 nm OD was measured and cells were centrifuged. Pellets were conserved at -80°C. After dilution with lysis buffer (volume adapted to the OD obtained) cells were lysed by sonication (10 minutes, 5s pulse, 50%

[1] Larentis AL, Argondizzo APC, Esteves G dos S, Jessouron E, Galler R, Medeiros MA. Cloning and optimization of induction conditions for mature PsaA (pneumococcal surface adhesin A) expression in Escherichia coli and recombinant protein stability during long-term storage. *Protein Expr Purif,* **2011** Jul;78(1):38–47.

amplitude). For each production, total cell lysis (first column) and supernatant after centrifugation (second column) were loaded on a SDS-PAGE gel (**Supplementary Figure 1**).

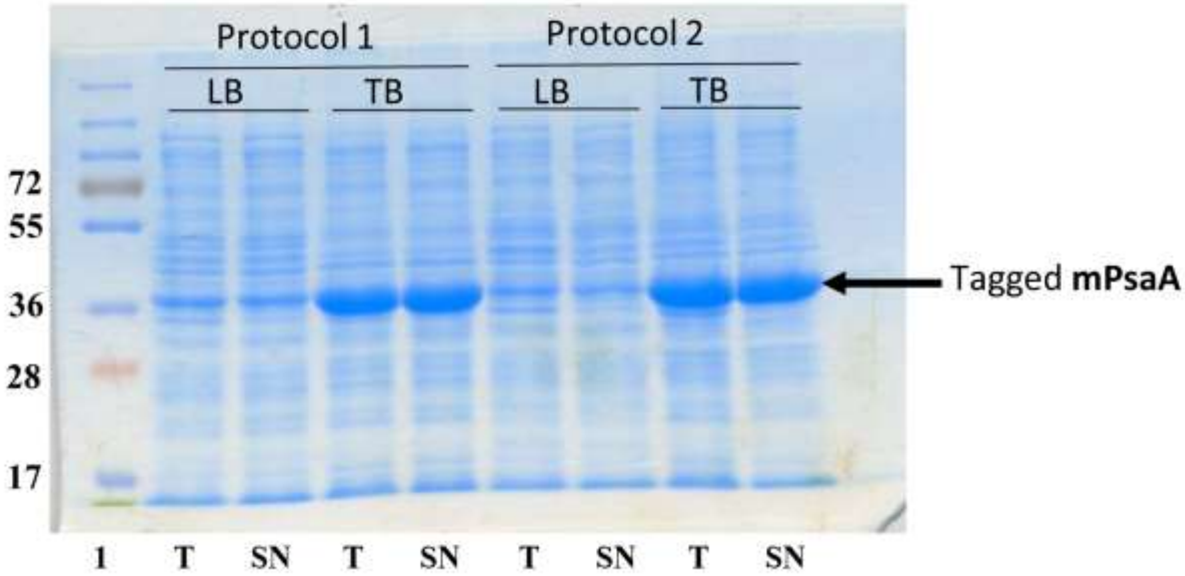

SUPPLEMENTARY FIGURE 1. Analysis mPsaA production following the four different culture growth conditions by SDS-PAGE. Lane 1: unstained protein markers. T = total cell lysis fraction; SN = Supernatant lysis fraction.

There is no significant difference in protein expression profil between the total cell lysis fraction and the supernatant after centrifugation for a given production. A band corresponding to the tagged mPsaA is visible for every production. However, the band corresponding to the tagged mPsaA is more intense when the bacteria are grown in TB compared to the LB media independently of the considered protocol indicating that the former medium is superior in inducing overexpression of the mPsaA.

Total and tagged mPsaA protein amounts were further quantified for each production using Bradford assays before and after metal-affinity chromatography purifications (NiNTA), respectively (**Supplementary Table 1**).

Supplementary Table 1: Amount of protein and tagged mPsaA produced per liter of culture

	**LB Protocol 1**	**TB Protocol 1**	**LB Protocol 2**	**TB Protocol 2**
**Total Protein (mg)**	375	1040	550	2110
**Tagged mPsaA (mg)**	10	50	15	100
**Ratio tagged mPsaA/protein**	2,7 %	4,8 %	2,7 %	4,7 %

From this table we can conclude that the use of TB medium leads to a 5 fold increase of tagged mPsaA production. The production can reach 100 mg/l when incubation is carried out at 25°C during 16 hours after induction with 0.1 mM of IPTG in TB medium.

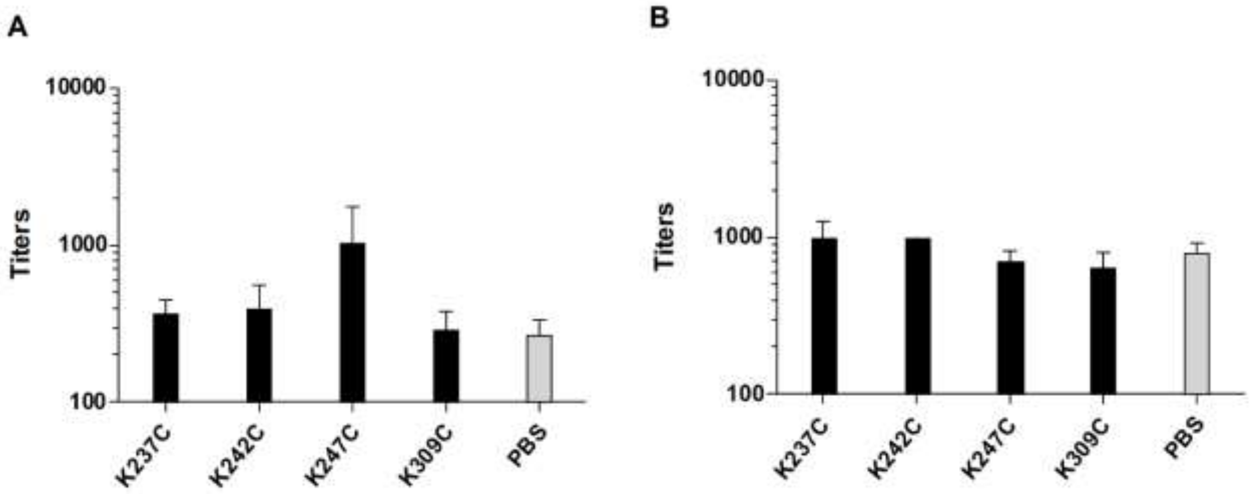

SUPPLEMENTARY FIGURE 2. Titers of anti-CP14 IgM Abs of mice immunized with Pn14TS-mPsaA K237C, K242C, K247C, K309C and PBS after the 2nd (A) and the 3rd immunization. The serum samples data presented as geometric mean titer ± standard deviation of five mice per group.

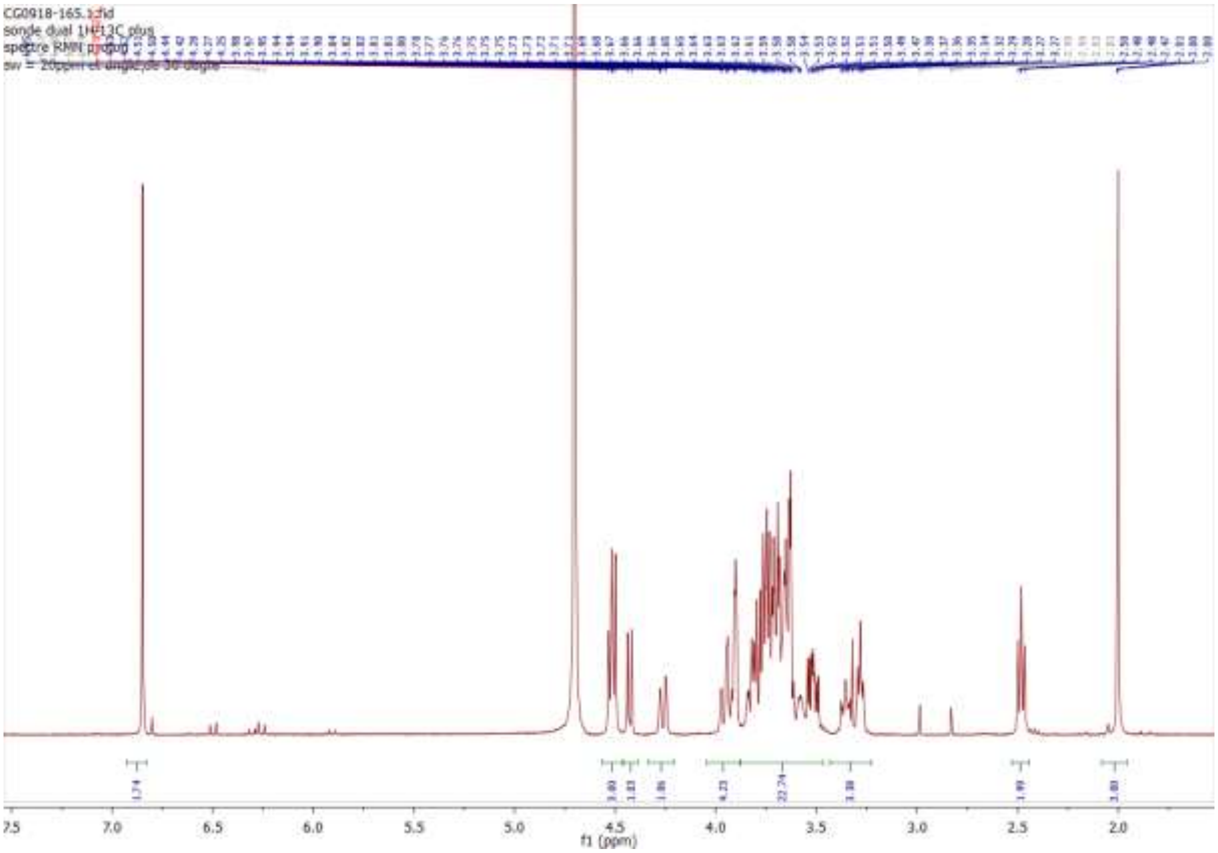
CG0918-165.1.fid
sonde dual 1H/13C plus
f1 (ppm)

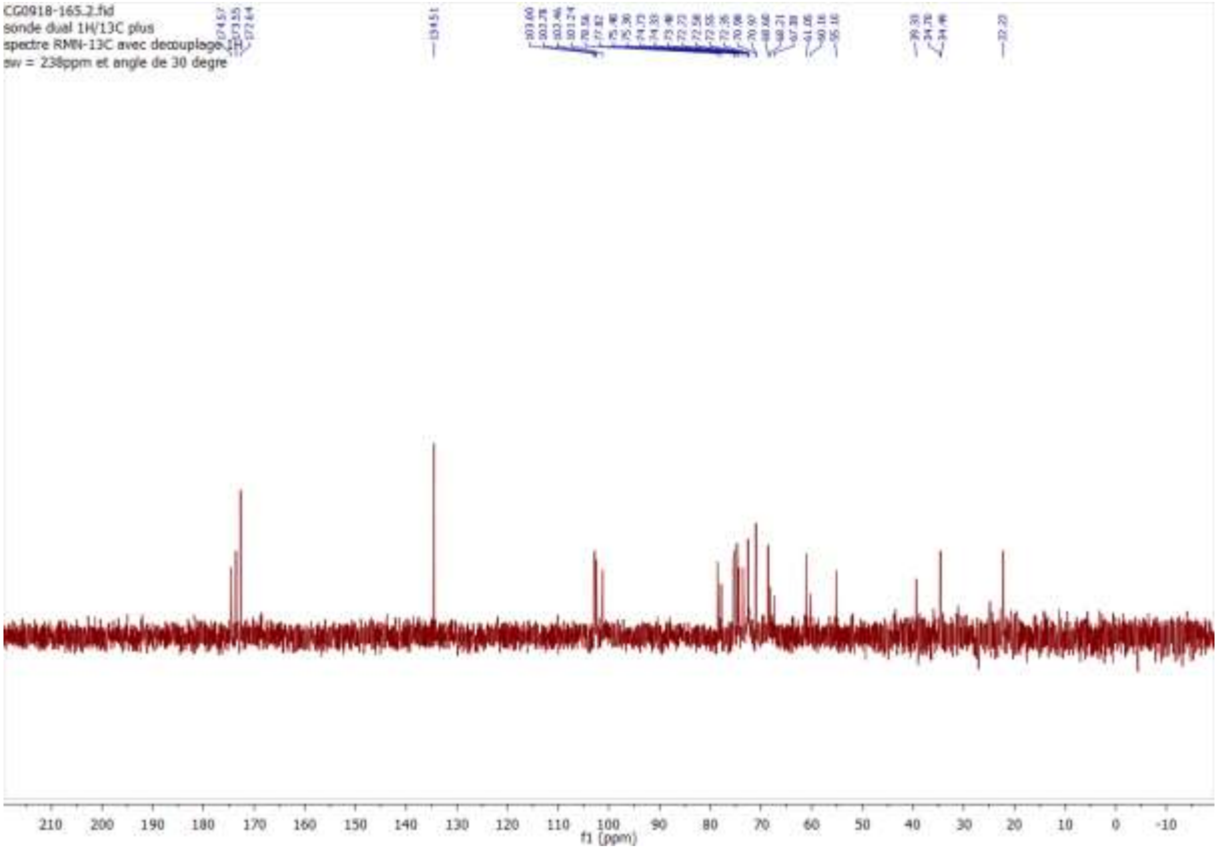
CG0918-165.2.fid
sonde dual 1H/13C plus
spectre RMN-13C avec decouplage 1H
sw = 238ppm et angle de 30 degre
f1 (ppm)
